# The Treatment of Delirium Tremens

**Published:** 1917-02-03

**Authors:** 


### THE DRUG AND ALCOHOL HABIT.

### The Treatment of Delirium Tremens.

THE tenth annual report of the Norwood Sanatorium has been issued very promptly, and is a model of what such documents ought to be.

Of the six cases of morphia habit admitted during the year, it is significant that five were in medical men, and the remaining one was the wife of a medical man. In four cases the habit was acquired owing to the administration of the drug for the relief of pain. In the fifth it was taken to relieve mental depression, and in the sixth was begun through mere curiosity, another illustration of the danger of playing with fire. The two cases of morphinism and cocainism combined were in medical students. In one the administration was begun for the relief of pain; in the other to procure sleep. One patient was admitted for the omnopon habit. This drug was resorted to as an aid to the relinquishment of alcohol, and was prescribed on account of its being reputed to be harmless. The result was to leave the patient addicted to both habits.

### WHAT DRIVES MEN TO ALCOHOL.

There was one case of the heroin habit. It is characteristic also that one case in which numerous drugs were taken was that of a young woman of twenty-five who had acquired the habit of taking morphia when she was a hospital nurse.

Among the alcoholic cases, several patients blamed their occupations for their habits. In two cases alcohol was taken to relieve nerve shock inflicted at the Front. Want of occupation and boredom was the originating factor in two cases, both in men who had made a competency by hard work, had sold out, and retired. Financial worry and loss were responsible in three cases. In five cases a husband has been driven to drink by the trying temper or habits of his wife, and in two cases alcohol was taken to relieve the distress caused by a sudden death in the family. In the other cases the causes were various. In three alcohol was taken to relieve some painful or distressing affection. Three of the patients blamed the fact that they began to drink beer when at school, and in about twenty cases moderate drinking had slowly but progressively increased to drunkenness.

Among the complications probably due to alcoholism were insomnia in five cases; extreme loss of memory in two cases; mental depression and insanity in two cases; peripheral neuritis in ten cases; epilepsy in one case; dyspeptic gastric catarrh in five cases; and among the remainder two cases of delirium tremens.

Dr. Hare again emphasises and reinforces his opinion, which cannot any longer be contested, that delirium tremens is due, not to taking alcohol, but to the sudden deprivation of alcohol from those who are accustomed to taking large quantities. He has shown again and again that this factor is never absent in the immediate past of delirium tremens. Usually the alcohol has been suddenly reduced or altogether stopped on medical advice, and there is no doubt that the practice of forbidding altogether the taking of alcohol by persons who are accustomed to take it in large quantities is responsible for the majority of cases of delirium tremens. In other cases the alcohol is cut off by accident. A man breaks his leg and is carried into hospital, and within two or three days he becomes the victim of delirium tremens. In such cases it is usual to ascribe the delirium to the shock of the accident acting upon an alcoholised subject. If it could be shown that delirium tremens followed an accident of the same kind in cases in which the alcohol continued to be taken in the usual quantity, such an hypothesis would be plausible; but there is no case on record in which this has happened. When a man breaks his leg and is confined to bed, he is almost certain to be at the same time deprived of his alcohol. If he is taken into a hospital, he cannot get it; if he is put to bed at home, he either—having been a secret drinker—voluntarily deprives himself of it, or his relatives think it a good opportunity to compel him to break off the habit, or his doctor takes the opportunity to do the same thing. In every case in which delirium tremens occurs, alcohol has been cut off. As we know that delirium tremens may be thus caused, it is very unscientific to call in aid causation by shock, of the existence of which there is often no evidence, and of the efficacy of which there is never any evidence. There is a small residue of cases in which delirium tremens occurs in patients who continue to drink their excessive quantity of alcohol, but in such cases it is invariably found that the alcohol, although it is imbibed, is not absorbed; for the patient suffers from vomiting, due to the gastritis that the alcohol has set up; or due to nervous origin dependent on the action of alcohol on the nervous system.

"Patients," says Dr. Hare, "who have for some time been drinking spirits heavily and continuously are, as far as I can judge, always on the brink of vomiting, which once started may become practically uncontrollable."

The proof of the pudding is in the eating, and the proof that delirium tremens is due to the sudden deprivation of alcohol is shown by the fact that the attack of delirium can be cut short in an early stage by administration of the normal quantity of alcohol. One such case is described in the report under consideration.

